# Discordance Between Thyroid Function and Thyroid-Stimulating Hormone (TSH) Receptor Antibodies in Down Syndrome Patients With Autoimmune Thyroid Disease: A Long-Term Follow-Up Study of Two Cases

**DOI:** 10.7759/cureus.78457

**Published:** 2025-02-03

**Authors:** Yuki Shibagaki, Shigeru Suzuki, Akiko Furuya, Takahide Kokumai, Satoru Takahashi

**Affiliations:** 1 Pediatrics, Asahikawa Medical University, Asahikawa, JPN

**Keywords:** down syndrome(ds), graves´disease, hashitoxicosis, tsh receptor antibody, tsh receptor stimulating antibody, tsh-stimulating blocking antibody, hashimoto’s thyroiditis

## Abstract

Thyroid function in autoimmune thyroid disease (AITD) among patients with Down syndrome (DS) sometimes exhibits fluctuations over time, but the underlying cause of this variability remains unknown. Thyrotoxicosis is often associated with Graves disease (GD) and Hashitoxicosis. Thyroid-stimulating hormone (TSH) receptor antibody (TRAb) includes thyroid-stimulating antibody (TSAb) and TSH-stimulation blocking antibody (TSBAb), causing GD or hypothyroidism, respectively. We report two patients of DS showing discordance between thyroid function and TRAbs. Case 1: A 16-year-old boy with DS was diagnosed with Hashimoto thyroiditis at age 6, with positive TSAb and TSBAb. At age 7, GD was diagnosed based on thyrotoxicosis and elevated I^123^ uptake. His TRAb became negative after 18 months of methimazole treatment. During follow-up, recurrent thyrotoxicosis with positive TRAb/TSAb improved transiently without intensified treatment. Methimazole was discontinued at age 14, but hypothyroidism with positive TSAb and negative TSBAb became evident. Case 2: An 18-year-old woman with DS was diagnosed with Hashimoto thyroiditis at age 13. She experienced recurrent transient thyroiditis with positive TRAb/TSAb. Although TSAb remained positive and TSBAb negative, hypothyroidism continued without GD. In conclusion, our findings indicate that awareness of the potential discordance between TSAb/TSBAb status and thyroid function is important for deciding appropriate treatment in DS patients with unstable thyroid function and AITD.

## Introduction

Thyroid disease is a common complication in patients with Down syndrome (DS), with a higher prevalence compared to that in the general population, estimated at approximately 24-28% [1,2]. Among these, the clinical course of autoimmune thyroid disease (AITD), such as Hashimoto thyroiditis (HT) and Graves disease (GD), has often been reported to differ from that of the general population [3-5].

HT, the most common cause of hypothyroidism, is diagnosed based on the presence of diffuse thyroid gland enlargement (or, less commonly, atrophy) and anti-thyroid peroxidase autoantibodies (TPOAb) and/or anti-thyroglobulin antibody (TgAb) [6]. While cytological evidence of lymphocytic infiltration can provide a definitive pathological diagnosis, it is not necessary for clinical diagnosis. Importantly, HT can manifest as euthyroidism (normal thyroid function), hypothyroidism (characterized by insufficient thyroid hormone production leading to symptoms such as fatigue, weight gain, and cold intolerance), or thyrotoxicosis (characterized by excess thyroid hormone leading to symptoms such as tachycardia, weight loss, and increased sweating) due to destructive thyroiditis (also known as Hashitoxicosis). Hashitoxicosis results from the release of preformed thyroid hormones due to thyroid follicle destruction, showing decreased uptake on thyroid scintigraphy as the gland is not overactive. This thyrotoxic phase typically resolves within two to three months as inflammation subsides, with thyroid function returning to either an euthyroid or hypothyroid state. In contrast, GD is a common cause of hyperthyroidism-induced thyrotoxicosis [7]. In GD, hyperthyroidism results in excessive thyroid hormone production and secretion. GD is diagnosed when thyrotoxic symptoms, and diffuse thyroid enlargement are observed, along with elevated free T3 (FT3) or free T4 (FT4) levels, suppressed thyroid-stimulating hormone (TSH), positive TSH receptor antibody (TRAb) or thyroid-stimulating antibody (TSAb), and characteristic findings on thyroid scans, such as 123I and 99mTcO4 thyroid scintigraphy. It is important to note that both hypothyroidism and thyrotoxic conditions can be diagnosed through screening tests in asymptomatic individuals [6,7].

HT in DS occurs at an early age, while hypothyroidism often appears as a complication or progression of HT. Furthermore, cases have been reported where it progresses to GD [3]. In terms of GD, there are reports of both good responses to treatment [4] and refractoriness [5], the reasons for which are unknown. Hashitoxicosis is reported to be an infrequent cause of thyrotoxicosis in patients with DS [5]. Owing to significant differences in treatment approaches, GD must be differentiated from Hashitoxicosis [7]. While GD requires antithyroid medication to manage the hyperthyroid state, Hashitoxicosis typically resolves spontaneously without such intervention. In fact, administering antithyroid drugs in cases of Hashitoxicosis is unnecessary and would expose patients to potential side effects without therapeutic benefit.

It is useful in differential diagnosis to measure the levels of TRAb, which are usually present in GD and have a high sensitivity and specificity for its diagnosis [8]. There are two types of TRAbs: TSAb, which has a stimulating effect, and TSH-stimulation blocking antibody (TSBAb), which exerts an inhibitory effect. TRAb, measured as TSH-binding inhibitory immunoglobulin, indicates the inhibition of TSH binding to TSH receptor (TSHR) but does not provide information about the functional properties of TRAb. To determine whether TRAb is stimulatory or inhibitory, bioassays are used to measure TSAb and TSBAb. TSAb is measured by assessing cAMP production in thyroid cell linens transfected with TSHR, while TSBAb is measured by evaluating the inhibition of cAMP production following TSH-mediated stimulation of TSHR-transfected cells [9]. The TRAb involved in the pathogenesis of GD is TSAb. In cases of thyrotoxicosis that are negative for TRAb and TSAb, a diagnosis of GD is typically ruled out [8,10]. TSBAb is found in some patients with HT or atrophic thyroiditis, and TSBAb causes hypothyroidism [11]. Herein, we describe two cases of DS with AITD in which TRAb, TSAb, and TSBAb levels could not expect thyroid dysfunctions during the clinical course.

## Case presentation

Case 1

**Figure 1:**
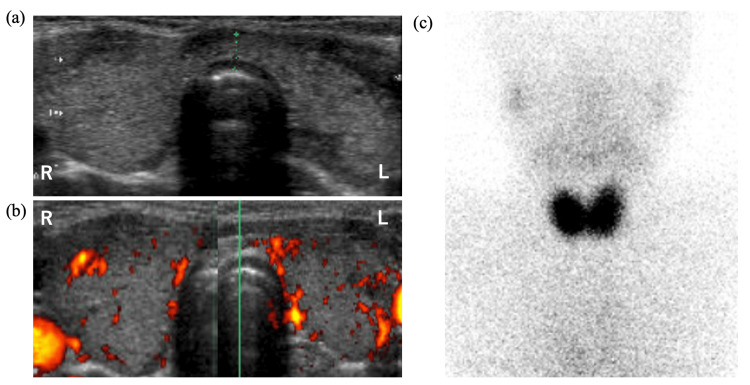
Ultrasound images of B-mode (a) and color Doppler (b) at six years of age when Hashimoto thyroiditis (HT) was diagnosed and a thyroid scan with 99mTc (c) at seven years of age when Graves disease (GD) was diagnosed in case 1.

**Figure 2:**
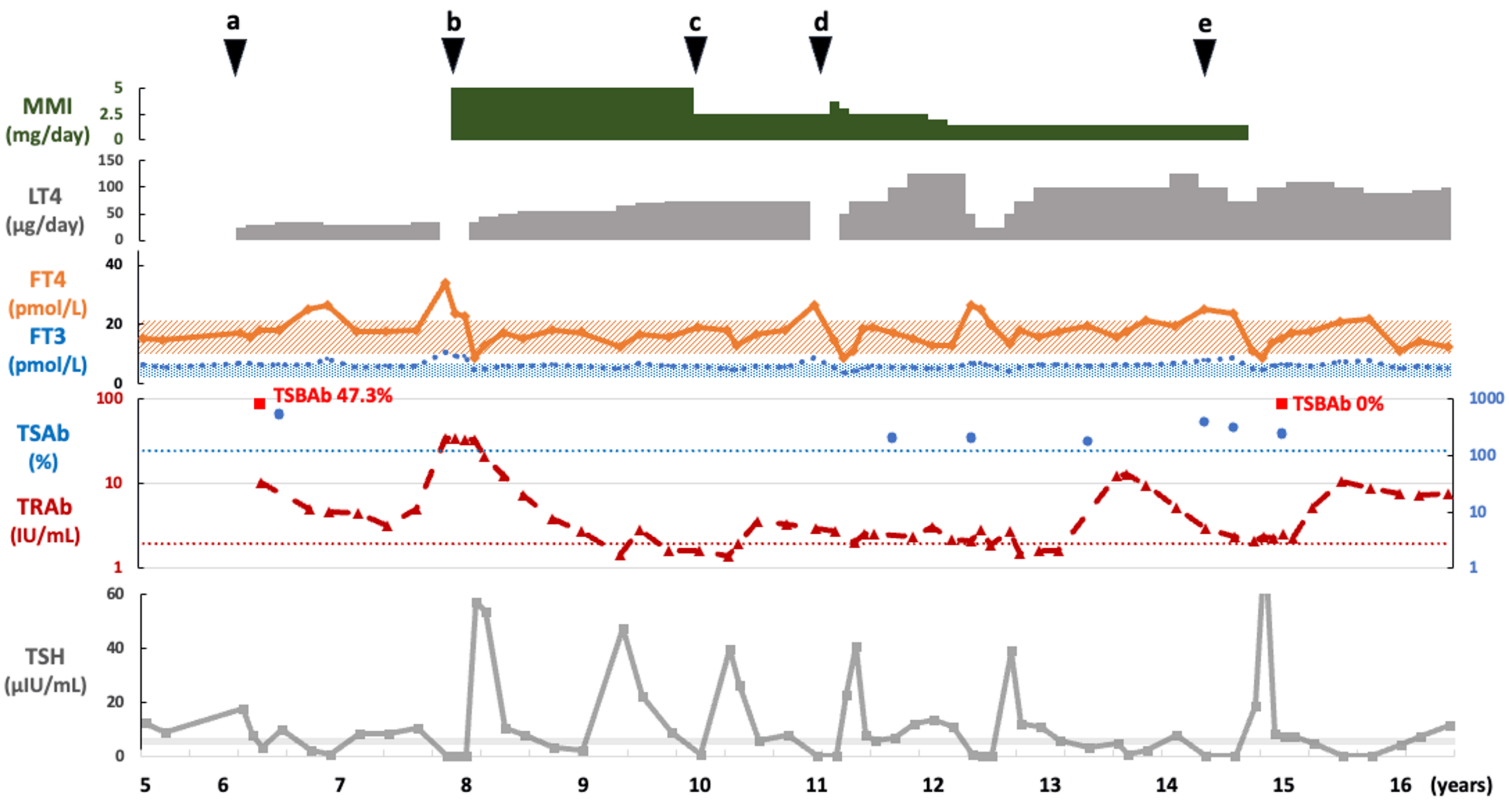
Case 1: The changes in thyroid function and course of treatment. The patient was diagnosed with HT at age 6 (black arrow a) and with GD at age 7 (black arrow b). TRAb was positive, and LT4 was discontinued while MMI was started. At age 10, a follow-up TRAb test was negative; thus, MMI dosage was reduced (black arrow c). At age 11, the patient developed TRAb-positive thyrotoxicosis, but his condition progressed to hypothyroidism one month later, and Hashitoxicosis was suspected (black arrow d). At age 14 years and five months, TgAb and TPOAb levels were elevated compared to the initial visit. Thyroid ultrasound showed characteristics of HT, and methimazole therapy was subsequently discontinued (black arrow e). FT3, free T3; FT4, free T4; GD, Graves disease; HT, Hashimoto thyroiditis; LT4, levothyroxine; MMI, methimazole; TgAb, anti-thyroglobulin antibody; TPOAb, thyroid peroxidase autoantibodies; TRAb, TSH receptor antibody; TSAb, thyroid-stimulating antibody; TSBAb, TSH-stimulation blocking antibody

Our first case was a 16-year-old male who was born at 36 + 2 weeks gestation via emergency Caesarean section, weighed 2,098 g at birth, and was diagnosed with DS. There was no relevant family history, and his newborn screening results were normal. Complications included congenital duodenal stenosis and an atrial septal defect. He underwent surgery to treat the former five days following his birth, and the latter closed naturally. He later developed a goiter (Shichijo classification, grade 3 [12] and HT at age 6, when laboratory measurements revealed subclinical hypothyroidism, with an elevated TSH level of 17.49 μIU/mL (normal range, 0.50-5.00 µIU/mL), normal FT4 level of 14.9 pmol/L (normal range, 11.6-21.9 pmol/L), positive anti-TgAb level of 957 IU/mL (negative ≤28 IU/L), positive TPOAb level of 55.0 IU/mL (negative ≤16 IU/L). Ultrasonography of the thyroid revealed a goiter, swelling, and slightly elevated blood flow (Figures 1a-1b). The patient was diagnosed with HT, and treatment with levothyroxine (LT4) was initiated (Table 1). At the time of diagnosis, he was positive for autoimmune markers, with a TRAb level of 10.29 IU/mL (normal range, <2.0 IU/mL), TSBAb of 47.3% (normal range, <34%), and TSAb of 537% (normal range, <120%) (Table 1, Figure 2). After starting LT4, his TSH levels remained unstable and deviated from the normal range.

**Table 1:** The thyroid function test and TSH receptor antibodies at diagnosis case 1. FT3, free T3; FT4, free T4; N/A, not applicable; TgAb, anti-thyroglobulin antibody; TPOAb, thyroid peroxidase autoantibodies; TRAb, TSH receptor antibody; TSAb, thyroid-stimulating antibody; TSBAb, TSH-stimulation blocking antibody

Laboratory parameters	TSH	FT3	FT4	TgAb	TPOAb	TRAb	TSAb	TSBAb
Unit of measurement	(µIU/mL)	(pmol/L)	(pmol/L)	(IU/mL)	(IU/mL)	(IU/mL)	(%)	(%)
Normal range	0.50-5.00	3.5-6.1	11.6-21.9	<28	<16	<2.00	<120	<34.00
Case 1 (seven years old)	17.49	N/A	14.9	957	55	10.29	537	47.33

At the age of seven years, he developed GD. The diagnosis was supported by thyrotoxicosis indicated by thyroid function tests - a low TSH level of 0.04 µIU/mL, elevated FT3 of 10.8 pmol/L (normal range, 3.5-6.1 pmol/L), elevated FT4 of 33.9 pmol/L, along with a strongly positive TRAb level of 34.4 IU/L. Thyroid technetium scintigraphy, showing an elevated 20-minute 99mTc uptake of 3.4% (cutoff value, <1%; Figure 1c [12]), confirmed the diagnosis. LT4 was stopped, and 5 mg (0.27 mg/kg) of methimazole was started. The patient’s thyroid function improved in a short period of time, approximately two months; however, due to high TRAb levels, the dose of methimazole was not reduced, and LT4 was administered concurrently (Figure 2). At the age of 10, a follow-up TRAb test was negative; hence, methimazole was reduced to 2.5 mg. At age 11, he was diagnosed with thyrotoxicosis and found to be positive for TRAb; therefore, his methimazole dose was increased to 3.75 mg. However, his condition progressed to hypothyroidism just one month later. Since it was possible that this was a case of Hashitoxicosis rather than a recurrence of GD, methimazole was again reduced to 2.5 mg. Despite gradually increasing the LT4 dose to 125 µg, subclinical hypothyroidism persisted, and methimazole was therefore reduced to 1.5 mg. At the age of 14 years and five months, TgAb (2,778 IU/mL) and TPOAb (494 IU/mL) levels were elevated compared to the initial visit. Thyroid ultrasound showed mild enlargement, an irregular surface, and heterogeneous internal echogenicity, consistent with HT. Subsequently, methimazole therapy was discontinued. At the ages of 12 years, six months, and 14 years, four months, although he transitioned to thyrotoxicosis with positivity for both TRAb and TSAb, he developed subclinical hypothyroidism within one month without any increase in his methimazole dose. Based on this rapid improvement in thyroid function, we diagnosed what appears to be Hashitoxicosis, although thyroid scintigraphy could not be performed because thyroid function normalized before the test could be conducted. At the age of 14 years and five months, methimazole therapy was discontinued. Despite continued positivity for TRAb and TSAb and negativity for TSBAb, his hypothyroidism has persisted.

Case 2

Our second case was an 18-year-old woman who was born at 39 + 5 weeks of gestation via vaginal delivery, weighed 2,490 g at birth, and was diagnosed postnatally with DS. There was no relevant family history, and newborn screening results were normal. She experienced complications, including duodenal atresia and patent ductus arteriosus. The former was surgically treated at two days following her birth, whereas ductus arteriosus ligation was performed at 10 months to treat the latter.

At the age of 10 years, laboratory measurements revealed that the patient tested positive for TgAb (124 IU/mL) and TPOAb (137 IU/mL). At age 13, a palpable goiter was found (Shichijo’s classification [12], grade 2), and thyroid function studies revealed that the patient had subclinical hypothyroidism, with an elevated TSH level of 26.03 µIU/mL, normal FT3 level of 4.7 pmol/L, normal FT4 level of 10.6 pmol/L, and positivity for TPOAb at 148 IU/mL. She was diagnosed with HT, and treatment with LT4 was initiated (Table 2).

**Table 2:** The thyroid function test and TSH receptor antibodies at diagnosis case 2. FT3, free T3; FT4, free T4; N/A, not applicable; TgAb, anti-thyroglobulin antibody; TPOAb, thyroid peroxidase autoantibodies; TRAb, TSH receptor antibody; TSAb, thyroid-stimulating antibody; TSBAb, TSH-stimulation blocking antibody

Laboratory parameters	TSH	FT3	FT4	TgAb	TPOAb	TRAb	TSAb	TSBAb
Unit of measurement	(µIU/mL)	(pmol/L)	(pmol/L)	(IU/mL)	(IU/mL)	(IU/mL)	(%)	(%)
Normal range	0.50-5.00	3.5-6.1	11.6-21.9	<28	<16	<2.00	<120	<34.00
Case 2 (13 years old)	26.03	4.7	10.6	N/A	148	N/A	N/A	N/A

**Figure 3:**
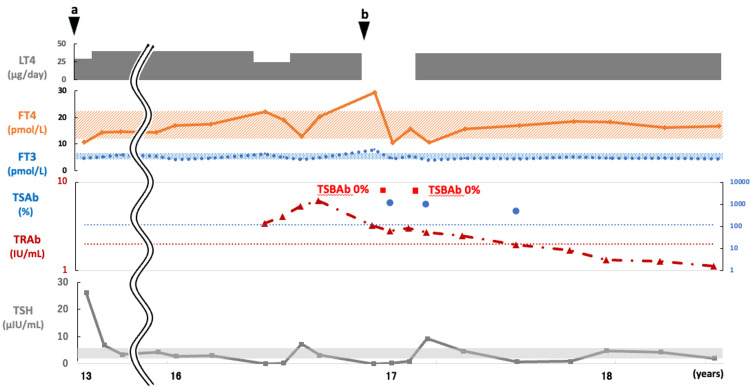
Case 2: The changes in thyroid function and course of treatment. The patient was diagnosed with HT at age 13 (black arrow a). At the age of 16, recurrent thyrotoxicosis with positive TRAb was observed, but the condition improved without methimazole, leading to a diagnosis of suspected Hashitoxicosis (black arrow b). TSBAb was consistently negative. FT3, free T3; FT4, free T4; HT, Hashimoto thyroiditis; LT4, levothyroxine; TSAb, thyroid-stimulating antibody; TRAb, TSH receptor antibody; TSBAb, TSH-stimulation blocking antibody

The patient’s thyroid function and treatment progression are shown in Figure 3. After treatment with LT4, her thyroid function stabilized. At the age of 16, recurrent thyrotoxicosis with positive TRAb was observed. TSAb was also positive, but the condition improved without treatment, leading to a potential diagnosis of Hashitoxicosis. 99mTcO4 thyroid scintigraphy was not performed because thyroid function improved rapidly before the test could be conducted. Subsequently, while still positive for TRAb and TSAb, the condition shifted to hypothyroidism, although TSBAb was negative. The period of positivity for TRAb and TSAb persisted, but GD did not develop.

## Discussion

The present study reported the long-term follow-up of two cases of DS with AITD and indicated that the discrepancies between the anti-thyroid antibodies and thyroid function may underline the variable course of their thyroid functions. In case 1, both TSAb and TSBAb were present at the time of hypothyroid with HT diagnosis, which was followed by GD. In the course of GD, possible Hashitoxicosis accompanied by conversion of TRAb positivity with high TSAb occurred, whereas recurrence of GD was unlikely because it improved in a short period of time without treatment escalation. After the transition to GD, the patient developed hypothyroidism despite his TSBAb levels becoming negative, but TSAb remained positive. In case 2, the transient state of elevated FT3 and FT4 with suppressed TSH was highly suggestive of Hashitoxicosis despite the presence of TRAb and TSAb. TSAb remained positive throughout the course, while TSBAb was negative; however, the patient remained hypothyroid but did not develop GD (Table 3).

**Table 3:** Changes in thyroid function and anti-thyroid antibodies of cases 1 and 2. GD, Graves disease; HT, Hashimoto thyroiditis; N/A, not applicable; TRAb, TSH receptor antibody; TSAb, thyroid-stimulating antibody; TSBAb, TSH-stimulation blocking antibody

Laboratory parameters	Case 1			Case 2		
Clinical course	At onset	During follow-up	At last visit	At onset	During follow-up	At last visit
Thyroid function	Hypothyroid	Thyrotoxicosis	Hypothyroid	Hypothyroid	Thyrotoxicosis	Hypothyroid
Diagnosis	HT	GD followed by Hashitoxicosis	HT	HT	Hashitoxicosis	HT
Antibody status						
TRAb	+	+	+	+	+	-
TSAb	+	+	+	N/A	+	+
TSBAb	+	N/A	-	N/A	-	-

Distinguishing between GD and Hashitoxicosis is crucial, owing to the significant differences in treatment between these two conditions. In GD, anti-thyroid medication is necessary, whereas, in Hashitoxicosis, thyrotoxicosis naturally improves within a few months [6,7]. Although TRAb and TSAb represent useful indicators for the identification of GD, about 10% of patients with Hashitoxicosis are also positive for either of the antibodies [9], but there were no patients with Hashitoxicosis with both positive tests [10]. In contrast, our two patients were strongly suspected of having Hashitoxicosis based on their clinical course despite having positive TSAb and TRAb results or negative TSBAb results. These results suggest that thyrotoxicosis accompanied by an increase in TRAb or TSAb during GD does not necessarily indicate the relapse but should consider the possibility of Hashitoxicosis.

Thyroid scintigraphy is a definitive test for confirming the diagnosis of atypical GD [7,13]. Actually, in case 1, the diagnosis was confirmed using 99mTcO4 thyroid scintigraphy. However, facilities capable of performing nuclear medicine tests are limited, and it is often impractical to conduct them immediately. Therefore, as an alternative index for differentiation, an elevated FT3/FT4 ratio has been reported to be helpful when diagnosing GD (mean ratio, approximately 3.1-3.2 in GD vs. 2.3-2.9 in painless thyroiditis (including Hashitoxicosis)) [14]. Nevertheless, there is an overlap between these two diseases. In fact, the FT3/FT4 ratio at the time of GD diagnosis in case 1 was 2.7, while during possible Hashitoxicosis, it ranged from 2.3 to 3.1. Therefore, in cases of thyrotoxicosis complicated by HT, careful observation of the clinical course is advisable, even if patients are positive for both TRAb and TSAb.

Next, we discuss the significance of TRAb in HT. In some cases of HT, TSBAb is observed, which is also immunologically measured as TRAb [11]. TSBAb contributes to thyroid dysfunction, and a switch from TSBAb to TSAb, or changes in their relative balance, have been reported during the transition from TRAb-positive HT to GD or vice versa [11,15]. In our case 1, TSBAb and TSAb coexisted at the time of HT diagnosis, and it was assumed that TSBAb predominated over TSAb, resulting in hypothyroidism. The transition from hypothyroidism due to HT to GD was thought to be accompanied by a shift to a TSAb-dominant serology, as previously reported [15]. By contrast, the transition from GD to hypothyroidism in our case 1 did not involve TSBAb, and TSAb positivity persisted. Case 2 remained hypothyroidism, albeit with TSAb-positive and TSBAb-negative states. Therefore, hypothyroidism with a positive TSAb test in the present cases supports the idea that the inflammation of thyroid cells caused by HT may prevent these cells from responding to the stimulatory effect of TSAb [15].

In both of our cases, the patients experienced recurrent episodes of thyrotoxicosis and hypothyroidism, requiring adjustments in the doses of LT4 and methimazole. These cases exhibited the characteristic instability of AITD associated with DS, as previously reported in the literature [3-5]. In addition, this report highlights the potential role of discrepancies between anti-thyroid antibody levels and clinical presentations as a significant factor in the fluctuating course of AITD in these patients.

The most significant limitation of this study is the inability to perform thyroid scintigraphy, which is the most useful tool for differentiating between GD and Hashitoxicosis in all thyrotoxic episodes except for the initial thyrotoxicosis in case 1. However, the rapid progression to TSBAb-negative hypothyroidism without intensified treatment strongly suggests Hashitoxicosis. Nevertheless, it is important to note that the sensitivity of thyroid autoantibody assays, including TSBAb, is not always high, which limits our ability to definitively prove thyroid function fluctuations due to imbalances between TSAb and TSBAb. This constraint represents another limitation of our study. Additionally, the second limitation is the small number of patients who are retrospectively described. A prospective study comparing the presence of TRAb and the clinical course in a large cohort of individuals with DS-associated AITD to those without DS will elucidate the unique characteristics of AITD in DS patients, leading to the proposal of treatment guidelines specific to this population.

## Conclusions

The longitudinal analysis of the two cases of DS and AITD with unstable thyroid function showed that the relationship between TSAb/TSBAb status and thyroid function is discordant. Even in thyrotoxicosis with positive TRAb and TSAb during treatment of GD, Hashitoxicosis should be considered. Moreover, patients with DS can develop hypothyroidism followed by thyrotoxicosis even if TSAb remains positive and TSBAb is negative. Thus, the awareness of the discordance between TSAb/TSBAb status and thyroid function is important in deciding the appropriate treatment in DS patients with unstable thyroid function and AITD.

## References

[1] Pierce MJ, LaFranchi SH, Pinter JD (2017). Characterization of thyroid abnormalities in a large cohort of children with Down syndrome. Horm Res Paediatr.

[2] Tüysüz B, Beker DB (2001). Thyroid dysfunction in children with Down's syndrome. Acta Paediatr.

[3] Aversa T, Salerno M, Radetti G (2015). Peculiarities of presentation and evolution over time of Hashimoto's thyroiditis in children and adolescents with Down's syndrome. Hormones (Athens).

[4] De Luca F, Corrias A, Salerno M (2010). Peculiarities of Graves' disease in children and adolescents with Down's syndrome. Eur J Endocrinol.

[5] Goday-Arno A, Cerda-Esteva M, Flores-Le-Roux JA, Chillaron-Jordan JJ, Corretger JM, Cano-Pérez JF (2009). Hyperthyroidism in a population with Down syndrome (DS). Clin Endocrinol (Oxf).

[6] Caturegli P, De Remigis A, Rose NR (2014). Hashimoto thyroiditis: clinical and diagnostic criteria. Autoimmun Rev.

[7] Chaker L, Cooper DS, Walsh JP, Peeters RP (2024). Hyperthyroidism. Lancet.

[8] Dwivedi SN, Kalaria T, Buch H (2023). Thyroid autoantibodies. J Clin Pathol.

[9] Hollenberg A, Wiersinga WM, Bartalena L, Feldt-Rasmussen U (2020). Hyperthyroid disorders. Williams Text of Endocrinology.

[10] Kamijo K (2018). Clinical application of TSAb (EIA). Jpn Thyroid Assoc.

[11] McLachlan SM, Rapoport B (2013). Thyrotropin-blocking autoantibodies and thyroid-stimulating autoantibodies: potential mechanisms involved in the pendulum swinging from hypothyroidism to hyperthyroidism or vice versa. Thyroid.

[12] Shichijo Shichijo, K K (1958). Simple goiter. J Jap Soc Intern Med.

[13] Uchida T, Suzuki R, Kasai T (2016). Cutoff value of thyroid uptake of (99m)Tc-pertechnetate to discriminate between Graves' disease and painless thyroiditis: a single center retrospective study. Endocr J.

[14] Yoshimura Noh J, Momotani N, Fukada S, Ito K, Miyauchi A, Amino N (2005). Ratio of serum free triiodothyronine to free thyroxine in Graves' hyperthyroidism and thyrotoxicosis caused by painless thyroiditis. Endocr J.

[15] Takasu N, Matsushita M (2012). Changes of TSH-stimulation blocking antibody (TSBAb) and thyroid-stimulating antibody (TSAb) over 10 years in 34 TSBAb-positive patients with hypothyroidism and in 98 TSAb-positive Graves' patients with hyperthyroidism: reevaluation of TSBAb and TSAb in TSH-receptor-antibody (TRAb)-positive patients. J Thyroid Res.

